# Identification and Functional Analysis of Candidate Effectors in the Sorghum Aphid, *Melanaphis sorghi*

**DOI:** 10.3390/insects17080779

**Published:** 2026-07-28

**Authors:** Yongbang Li, Zhiqiang Hu, Yating Guo, Bingjie Du, Jing Yu, Yu Zhang, Qianqian Du, Guangwei Li, Daowen Wang, Di Wu

**Affiliations:** 1 State Key Laboratory of High-Efficiency Production of Wheat-Maize Double Cropping, Center for Crop Genome Engineering, College of Agronomy, Henan Agricultural University, Zhengzhou 450046, China; 2 The Shennong Laboratory, Zhengzhou 450002, China

**Keywords:** *Melanaphis sorghi*, sorghum, proteomics, defense responses

## Abstract

The sorghum aphid (*Melanaphis sorghi*), a phloem-feeding insect pest, causes severe economic losses in sorghum-producing regions worldwide. During feeding, aphids secrete salivary proteins with potential effector functions that play pivotal roles in plant–insect interactions. However, relatively few secreted proteins and effectors have been characterized in *M. sorghi*. In this study, proteomic analysis of aphid-infested sorghum leaves identified 69 putative aphid-derived proteins. Bioinformatic analyses were subsequently used to predict signal peptides within these proteins. Among the five candidates with signal peptides, MsSP1 was further confirmed to be delivered into sorghum tissues during aphid feeding by immunoblotting using a specific anti-MsSP1 antibody. Functional characterization revealed that silencing the *MsSP1* gene in aphids by RNAi interference significantly reduced aphid fitness on sorghum. Moreover, transient expression of *MsSP1* in *Nicotiana benthamiana* suppressed cryptogein-induced cell death and hydrogen peroxide accumulation, indicating that MsSP1 interferes with plant immune responses. Collectively, these results suggest that MsSP1 may act as a virulence effector that enhances aphid performance while suppressing host defense. This study expands current knowledge of plant–aphid interactions and provides new insights that may facilitate the development of sustainable pest-management strategies.

## Simple Summary

The sorghum aphid (*Melanaphis sorghi*) is a highly destructive agricultural pest that causes substantial yield losses in sorghum. Damage results not only from phloem sap extraction but also from the transmission of multiple plant viruses throughout crop development. During feeding, aphids deliver salivary proteins into host tissues, where some function as effectors that manipulate plant immune responses and facilitate infestation. In this study, proteomic analysis identified a repertoire of aphid-derived proteins detected in aphid-infested sorghum tissues, followed by functional annotation and enrichment analyses. Among these proteins, MsSP1, which contains a predicted signal peptide, was further characterized and shown to influence aphid feeding performance and alter plant immune responses. These findings suggest that MsSP1 may functions as a virulence effector that promotes aphid colonization of sorghum. This work expands the catalog of candidate effectors in *Melanaphis sorghi* and provides new insights into the development of RNAi-based strategies for aphid control.

## 1. Introduction

The sorghum aphid, *Melanaphis sorghi*, is a globally important piercing-sucking pest that causes extensive damage to sorghum through colony establishment, nutrient depletion, virus transmission, and increasing insecticide resistance [1]. Like other phloem-feeding insects, aphids possess highly specialized stylets that penetrate the plant epidermis, navigate intercellular spaces, and ultimately reach the phloem to extract sap [2,3]. Throughout this process, aphids secrete both gelling and watery saliva into plant tissues. Gelling saliva forms a salivary sheath surrounding the stylet, facilitating stylet movement while providing mechanical support, lubrication, and protection from plant-derived chemical defenses. Meanwhile, watery saliva is released directly into plant cells and contains detoxification enzymes, antioxidants, digestive enzymes, and cell wall-degrading proteins that alter host physiology, suppress defense responses, and promote sustained feeding [4,5,6]. Various substances and compounds in the saliva are key determinants of plant–insect interactions [5,7,8].

Notably, many secreted proteins in insect saliva function as elicitors or effectors to manipulate plant defense pathways [9,10,11,12,13]. Some secreted proteins are recognized by host plant surface-localized receptors and induce plant defense response to resist insect infestation [14,15,16]. In contrast, other secreted proteins can suppress plant defense response in order to enhance the survival and fecundity of insects [17,18,19]. Characterizing such molecules is therefore essential for understanding insect–plant interactions and may facilitate the development of environmentally sustainable pest-management strategies. In addition, effector- or elicitor-based approaches can enhance plant resistance and potentially improve biological control by increasing the attractiveness of herbivore-infested plants to natural enemies.

Advances in high-throughput sequencing and proteomics have greatly accelerated the discovery of insect secreted proteins [20,21,22,23]. Using bioinformatic approaches, 324 putative secreted proteins were identified in *Acyrthosiphon pisum*, many of which appear to evolve more rapidly than homologous proteins in other insects [24]. Similarly, transcriptome-based analyses have predicted hundreds of salivary proteins across several aphid species, including the potato aphid, wheat aphid, and pea aphid [19,25,26]. However, it is still limited in distinguishing the actual secreted proteins from the transcriptome data, while liquid chromatography–tandem mass spectrometry (LC-MS/MS) provides a particularly direct and reliable means of identifying secreted proteins from piercing-sucking insects. Proteomic analyses of saliva from three aphid species revealed proteins associated with ATP binding and oxidoreductase activity that may contribute to defense suppression in host plants [14]. In the tea green leafhopper, LC-MS/MS identified 152 salivary proteins involved in feeding, development, and interactions with host defenses [27]. Likewise, proteomic analysis of saliva collected from artificial diets of the potato aphid (*Macrosiphum euphorbiae*) identified numerous phosphoproteins as well as three established aphid effectors, Mp1, Me10/Mp58, and Me23 [28]. Together, these studies demonstrate the value of secretome analyses for elucidating the composition and biological functions of salivary proteins in phloem-feeding insects.

Compared with pathogen effectors, insect effectors originate from multicellular eukaryotes and generally exhibit greater evolutionary conservation. Several aphid effectors have been identified across multiple species and retain conserved biological functions. For example, the *Macrosiphum euphorbiae* effector Me10 and its homologs from other aphids enhance aphid performance and alter host physiology [10,28,29]. Similarly, *Myzus persicae* Mp10 shares sequence similarity with insect chemosensory proteins and suppresses flg22-triggered immune responses [18]. The effector C002 and its putative orthologs are also highly conserved across at least five aphid species despite exhibiting distinct functions in different host plants [30,31]. These observations highlight the potential of conserved salivary effectors as targets for broad-spectrum aphid control strategies.

Although substantial progress has been made in identifying and characterizing aphid effectors, genomic and proteomic resources for *M. sorghi* remain limited. This knowledge gap is notable given the severe economic impact of this pest, which can reduce sorghum yields by 50–100% in affected regions, including China [32,33]. Considering other known interactions between aphids and host plants, we speculate that some secreted proteins in sorghum aphids may also function as key molecules to regulate sorghum defense. In this study, sorghum leaves infested with sorghum aphids were subjected to LC-MS/MS analysis to identify aphid-derived proteins that were delivered into host tissues. A total of 69 aphid proteins were detected, of which five contained predicted signal peptides and were selected for further investigation. Functional analysis identified MsSP1 (XP_025208241.1), a secreted protein of *M. sorghi*, as a candidate effector that enhances aphid performance on sorghum while suppressing host immune responses. By applying a proteomics-based strategy to identify candidate effectors in *Melanaphis sorghi*, this study provides important insights into the molecular mechanisms underlying aphid–sorghum interactions.

## 2. Methods

### 2.1. Plants and Insects

The susceptible sorghum genotype BTx623 was cultivated in a greenhouse maintained at 28 °C and 60% relative humidity under a 16 h light/8 h dark photoperiod. *Nicotiana benthamiana* plants were grown under similar conditions at 25 °C.

Colonies of sorghum aphid (*Melanaphis sorghi*) were maintained on one-month-old BTx623 plants in a growth chamber (28 °C, 60% relative humidity, and a 16 h light/8 h dark photoperiod) throughout the year. Sorghum aphid colonies were maintained clonally, and the inoculation, cultivation and observation of sorghum aphids in all the assays were conducted in the same environment.

### 2.2. Preparation of Protein Samples from Sorghum Leaves

BTx623 plants were grown in pots (10 cm diameter × 8.5 cm height). At the four-leaf stage, 20 aphid nymphs were confined to a designated leaf area using fine mesh cages for feeding for 48 h. Before sampling, the leaves were rinsed three times with ultrapure water to remove surface contaminants, including aphids, honeydew, exuviae and others. In total, 5 g of aphid-infested or non-infested control leaves were collected into one tube as one of three biological replicates. The samples were immediately frozen in liquid nitrogen, ground to a fine powder, and homogenized in lysis buffer containing 10 mM dithiothreitol and 1% protease inhibitor cocktail. An equal volume of Tris-saturated phenol (pH 8.0) was added, followed by centrifugation at 5000× *g* for 10 min at 4 °C. Proteins were precipitated overnight at −20 °C using ammonium sulfate-saturated methanol. The resulting protein pellet was dissolved in 8 M urea, and protein concentration was determined using a BCA protein assay kit.

For protein digestion, samples were precipitated with 20% trichloroacetic acid and incubated at 4 °C for 2 h. Following centrifugation at 4500× *g* for 5 min, the pellet was washed with pre-chilled acetone and resuspended in 100 mM TEAB. Proteins were reduced with 5 mM dithiothreitol at 56 °C for 0.5 h and alkylated with 11 mM iodoacetamide in the dark for 15 min. Samples were then digested overnight with trypsin at a 1:50 (*w*/*w*) trypsin-to-protein ratio.

### 2.3. Proteomic Analysis of Sorghum Leaves

Peptides were dissolved in 0.1% formic acid and 2% acetonitrile in water and separated using an EASY-nLC 1000 ultra-high-performance liquid chromatography system. Eluted peptides were analyzed with a timsTOF Pro mass spectrometer (Bruker Daltonics, Blyridge, MA, USA) equipped with a nano-electrospray ionization source and operated in parallel accumulation–serial fragmentation (PASEF) mode. Raw MS/MS data were processed using MaxQuant v1.6.15.0. Tandem mass spectra were searched against the *Sorghum bicolor* and *Melanaphis sacchari* protein databases (60,454 entries) concatenated with reverse decoy and contaminants database. Trypsin/P was specified as the cleavage enzyme, allowing up to 2 missing cleavages. The minimum length of the peptide segment was set to 7 amino acid residues, and the maximum number of modifications for the peptide segment was set to 5. The mass tolerances for precursor ions and fragment ions were both set to 20 ppm. Carbamidomethyl on Cys was specified as a fixed modification, and acetylation on the protein N-terminal and oxidation on Met were specified as variable modifications. The false discovery rate (FDR) for peptide and protein identification was set at <1%. Proteins with a minimum of one unique peptide were retained. Label-free quantitation (LFQ) intensity was employed to calculate the protein abundances. Proteins derived from aphids and those from sorghum were distinguished based on species-specific peptide sequences, as well as through protein grouping in MaxQuant and Razor assignment algorithms. To find proteins that were derived from aphids between aphid-infested samples and control samples, *t*-tests were used with the threshold set at *p*-value < 0.001, and the ratio was 1000. Signal peptides were predicted using the SignalP-5.0 Server (https://services.healthtech.dtu.dk/services/SignalP-5.0/, accessed on 5 July 2026) and SecretomeP (https://services.healthtech.dtu.dk/services/SecretomeP-2.0/, accessed on 5 July 2026). And the transmembrane regions were investigated through the TMHMM Server v2.0 (https://services.healthtech.dtu.dk/services/TMHMM-2.0/, accessed on 5 July 2026).

### 2.4. Cell Death Assay in N. benthamiana

The coding sequence of *MsSP1* without the signal peptide was amplified by PCR and cloned into a modified MYC-tagged pCAMBIA1300 vector to generate the MsSP1-MYC construct. The resulting plasmid was introduced into *Agrobacterium tumefaciens* strain GV3101. Agrobacterial cultures were grown overnight at 28 °C in LB medium supplemented with 50 μg/mL rifampicin and 25 μg/mL kanamycin. Collected cells were resuspended in infiltration buffer containing 10 mM MgCl_2_, 10 mM MES (pH 5.6), and 150 μM acetosyringone. After adjustment to OD_600_ = 0.6, suspensions were incubated at room temperature for 2 h and infiltrated into the leaves of four-week-old *N. benthamiana* plants using a 1 mL needleless syringe. Symptoms were monitored daily following infiltration.

The quantification of cell-death phenotype was measured by the electrolyte-leakage assay. Three leaf discs of the same size were placed into 6 mL of distilled water and incubated at room temperature for 3 h. The first conductivity (R1) was recorded using a conductivity meter. Subsequently, the second conductivity (R2) was tested after boiling for 30 min. Relative electrolyte leakage (R1/R2 × 100%) was calculated. The experiment was repeated three times.

For trypan blue staining, leaves were collected 5 days after infiltration and immersed in staining solution containing 10 g phenol, 10 mL glycerol, 10 mL lactic acid, 0.02 g trypan blue, and 10 mL water, as described by Lee et al. (2020) [34].

### 2.5. Immunoblot Analysis

For transient-expression assays, infiltrated tobacco leaves were harvested 48 h after inoculation. Total proteins were extracted from 500 mg leaf tissue using 500 μL extraction buffer containing 50 mM Tris-HCl (pH 7.5), 500 mM sucrose, 1 mM MgCl_2_, 10 mM EDTA, 0.1% NP-40, 5 mM DTT, and 1 mM PMSF. Samples were centrifuged at 12,000× *g* for 10 min at 4 °C, and supernatants were separated by 12% SDS-PAGE. Proteins were detected using anti-FLAG (M185-7; MBL) and anti-MYC (M192-3; MBL) antibodies.

Total proteins of sorghum protoplast were extracted by using the extraction buffer containing 100 mM Tris-HCl (pH 7.5), 5 mM MgCl_2_, 1 mM EDTA, 0.5% Triton X-100, 1 mM DTT, and 1 mM PMSF. Samples were shaken for 30 min and centrifuged at 12,000× *g* for 20 min at 4 °C. The resulting supernatant was separated by 12% SDS-PAGE and used for immunoblot analysis. Target proteins were detected using anti-FLAG (M185-7; MBL) and anti-GFP (G1102; LabLead; Beijing, China) antibodies.

To generate an anti-MsSP1 antibody, a specific MsSP1 polypeptide (SNAVPVKVSQSDQ) was synthesized and used for rabbit immunization (Mabnus, Wuhan, China). All procedures were approved by the Hubei Provincial Department of Science and Technology (SYSK-2021-0060, SCXK-2021-0011).

To verify the delivery of MsSP1 into host tissues, 20 aphid nymphs were confined to a defined region of BTx623 leaves using mesh cages for 48 h. Total proteins were extracted from aphid-infested leaves as described above and subjected to immunoblot analysis using the anti-MsSP1 antibody.

### 2.6. Subcellular Localization

The *MsSP1* coding sequence was amplified and cloned into the pRHVcGFP vector to construct the MsSP1-GFP recombinant plasmid. The MsSP1-GFP recombinant plasmid was co-transformed with the nucleus marker bZIP63-RFP [35] into BTx623 protoplasts, following the expression of fusion proteins (with GFP or RFP tag). The sorghum protoplasts were examined 24 h after transfection under a confocal microscope (Zeiss LSM980, Jena, Germany). GFP and RFP fluorescence signals were examined at 488 nm (emission at 507–550 nm) and 561 nm (emission at 588–630 nm), respectively. The Pearson correlation coefficient (r_P_) and Spearman correlation coefficients (r_S_) of colocalization were evaluated using Image J-java8 software.

### 2.7. DAB Staining and H_2_O_2_ Quantification

The constructs used in the above cell death assay were subsequently introduced into *A. tumefaciens* GV3101 and transiently co-expressed or individually expressed in *N. benthamiana* leaves for 36 h. Hydrogen peroxide accumulation was assessed by 3,3′-diaminobenzidine (DAB) staining as previously described [36]. Leaves were vacuum-infiltrated with DAB solution containing 1 mg/mL DAB, 10 mM MES (pH 3.8), and 0.2% (*v*/*v*) Tween-20 for 8–10 h. Chlorophyll was subsequently removed using 95% ethanol heated to 60 °C for 10 min. Cleared leaves were mounted in 50% glycerol and photographed.

For quantitative analysis, H_2_O_2_ content was measured using a hydrogen peroxide assay kit (Keming Biotechnology, Suzhou, China) according to the manufacturer’s instructions. Frozen leaf tissue (0.1 g) was ground in liquid nitrogen and homogenized in 1 mL extraction buffer. Following centrifugation at 8000 × *g* for 10 min at 4 °C, the supernatant was collected for analysis.

### 2.8. DsRNA Artificial Diet Bioassay

To minimize potential off-target silencing, the homologs of *MsSP1* in the *M. sorghi* genome were first checked, and no highly similar sequences of *MsSP1* were identified by BLAST search in NCBI (https://blast.ncbi.nlm.nih.gov/Blast.cgi, accessed on 3 June 2026). Target fragments of *MsSP1* and *GFP* were amplified using primers containing T7 promoter sequences. The primers used for dsMsSP1 synthesis were 5′-TAATACGACTCACTATAGGGCCATACTCCGTCCCACAACC and 5′-TAATACGACTCACTATAGGGTCGTCGAATGAGTGACCGTG, whereas dsGFP was amplified using primers 5′-TAATACGACTCACTATAGGGACCGAGAACAGGGTAAGTTGC and 5′-TAATACGACTCACTATAGGGACTTGGTGAACGTCCAGGAG. Purified PCR products served as templates for dsRNA synthesis using a MEGAscript RNAi Kit (Ambion, Austin, TX, USA). The resulting dsRNAs were verified by 1.2% agarose gel electrophoresis and quantified by absorbance at 260 nm.

For artificial diet assays, aphids collected from BTx623 plants were starved for 8 h before feeding. dsRNA was incorporated into a 20% sucrose artificial diet at a final concentration of 2.0 mg/mL, according to [37,38]. A 0.5 mL aliquot of diet was enclosed between two layers of Parafilm (2 cm diameter), and five aphid nymphs were placed on each feeding chamber. Feeding chambers were maintained in small glass dishes to prevent aphid escape, and survival was recorded daily. Diets containing dsGFP or no dsRNA served as controls. To evaluate RNAi efficiency, total RNA was extracted from five aphids per treatment after 48 h of feeding and analyzed by qRT-PCR. Subsequently, ten dsRNA-treated aphids were transferred to sorghum leaves, and aphid survival and reproduction were monitored daily.

Honeydew production was measured as described by Shangguan et al. (2018) [39] with minor modifications. Three-centimeter segments of one-month-old sorghum stems were placed in Petri dishes (2.5 cm diameter × 1 cm height) lined with filter paper. Three dsRNA-treated aphids were allowed to feed on the stems at 28 °C. Filter papers containing aphid excreta were collected and stained with 0.1% (*w*/*v*) ninhydrin in acetone at 2 days post-infestation. Following incubation at 60 °C for 30 min, honeydew deposits developed a violet-purple coloration. The stained area was quantified using ImageJ software. Each experiment was repeated three times.

### 2.9. Quantitative Real-Time PCR

Total RNA was extracted from dsRNA-treated aphids using TRIzol reagent (TaKaRa Bio, Shiga, Japan) according to the manufacturer’s protocol. First-strand cDNA was synthesized from 2 μg of total RNA using oligo(dT) primers and SuperScript II reverse transcriptase (Invitrogen, Beijing, China). Quantitative real-time PCR was performed using SYBR Premix Ex Taq™ II (TaKaRa Bio, Japan) on a Roche LightCycler 480 Real-Time PCR System with gene-specific primers qMsSP1-F and qMsSP1-R. The aphid *Actin* gene was used as an internal reference and amplified with primers qActin-F and qActin-R to normalize *MsSP1* transcript abundance. Primer sequences are listed in Table S4. All assays were conducted with at least three biological replicates.

### 2.10. Statistical Analysis

All experiments included three independent biological replicates, with each replicate having at least three technical repeats. The data was analyzed using Tukey’s HSD test with significance set at *p* < 0.05 in the Prism graphic software (Graph Pad Software, version 8.1.2, San Diego, CA, USA).

## 3. Results

### 3.1. Identification of M. sorghi Putative Secreted Proteins

**Figure 1:**
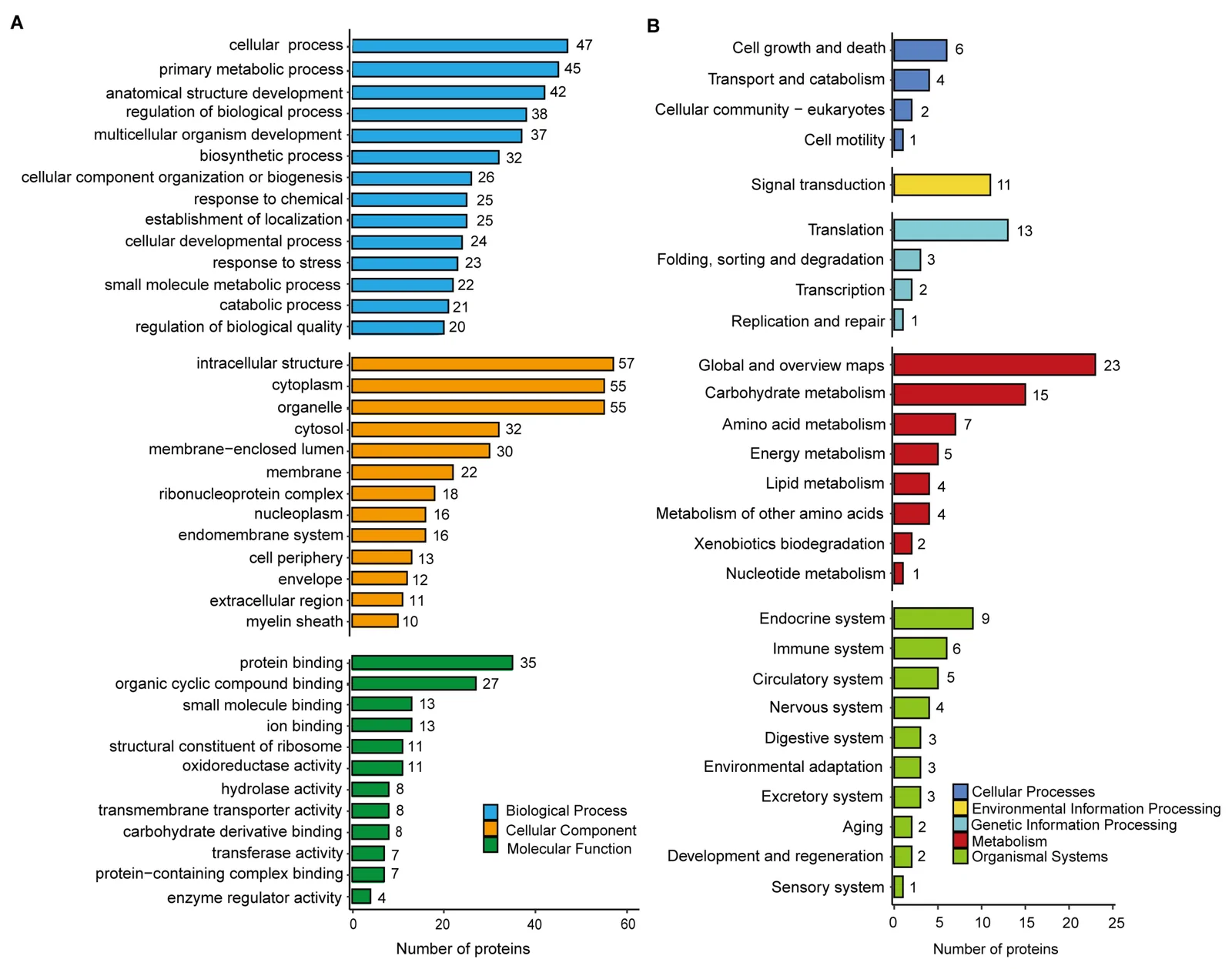
Functional annotation of *M. sorghi*-derived proteins. (**A**) Gene Ontology classification of these proteins at the second hierarchical level. Proteins were grouped into biological process, cellular component, and molecular function categories. (**B**) KEGG pathway classification of *M. sorghi*-derived proteins. Identified proteins were assigned to five major pathway groups: cellular processes, environmental information processing, genetic information processing, metabolism, and organismal systems.

Comparative LC-MS/MS analysis of aphid-infested and non-infested sorghum leaves identified 69 *M. sorghi*-derived proteins in aphid-infested sorghum tissues (Table S1). To characterize their potential functions, Gene Ontology (GO) and Kyoto Encyclopedia of Genes and Genomes (KEGG) enrichment analyses were performed. GO classification revealed that these proteins were predominantly associated with the biological processes of cellular process (47 proteins), metabolic process (45 proteins), and developmental process (42 proteins). Within the cellular component category, most proteins were assigned to intracellular anatomical structure (57 proteins), cytoplasm (55 proteins), and organelle (55 proteins). The major molecular function categories included protein binding (35 proteins), organic cyclic compound binding (27 proteins), and small-molecule binding (13 proteins) (Figure 1A, Table S2). KEGG pathway analysis grouped the identified proteins into five major functional classes: cellular processes, environmental information processing, genetic information processing, metabolism, and organismal systems (Figure 1B, Table S3). At the second hierarchical level, the most highly represented pathways were global and overview maps (23 proteins), carbohydrate metabolism (15 proteins), translation (13 proteins), signal transduction (11 proteins), and endocrine system pathways (9 proteins).

Because secreted proteins containing signal peptides are frequently considered candidate effectors in both pathogens and aphids [40,41,42], the aphid-derived proteins detected in aphid-infested sorghum tissues were further screened using SignalP 5.0. Five proteins were predicted to contain signal peptides (Table S1). The names of these five putative secreted proteins were assigned according to the closest matches in the National Center for Biotechnology Information (NCBI) database. XP_025201474.1 and XP_025204390.1 lacked functional annotation. XP_025200270.1 was annotated as a maltase A1-like protein, a protein family associated with carbohydrate and energy metabolism. XP_025203063.1 was identified as a lysine-rich arabinogalactan-like protein implicated in insect growth and development. XP_025208241.1 was annotated as a nuclear envelope pore membrane-like protein involved in nucleocytoplasmic macromolecular transport.

### 3.2. Sequence Analysis of the Candidate Protein MsSP1

**Figure 2:**
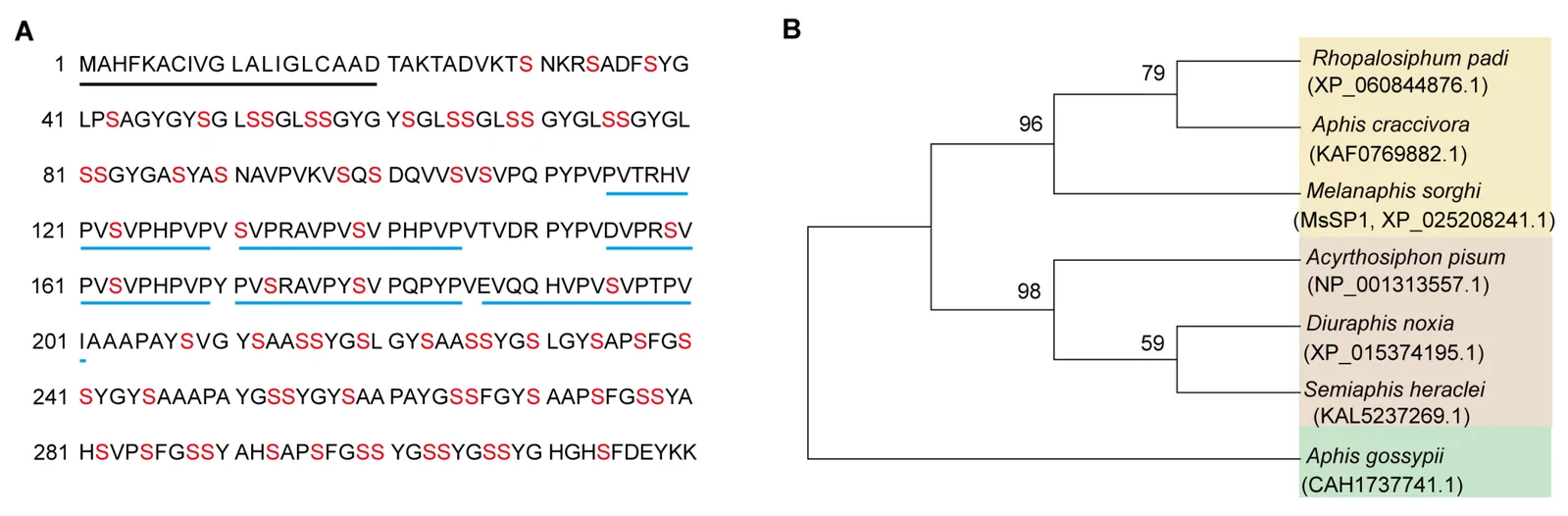
Amino acid sequence and phylogenetic analysis of MsSP1. (**A**) Amino acid sequence of MsSP1. The predicted signal peptide identified by SignalP is indicated by a black underline. Serine residues are highlighted in red. The five tandem repeats of the 15-amino-acid motif (PVPRAVPVSVPHPVP) are underlined in blue. (**B**) Phylogenetic relationships between MsSP1 and its orthologs from different aphid species. The phylogenetic tree was constructed using the neighbor-joining method in MEGA 11.

Nuclear envelope pore proteins mediate bidirectional transport between the nucleus and cytoplasm and are essential for the trafficking of regulatory molecules involved in growth, development, and immune signaling in eukaryotes [43,44,45]. XP_025208241.1, named MsSP1 (*Melanaphis sorghi* secreted protein 1), was subsequently selected for further sequence analysis and functional studies based on its annotation and abundance. Although no functional domain was detected by protein structure prediction, *MsSP1* encodes a 320-amino-acid protein containing a predicted N-terminal signal peptide spanning residues 1–20 and lacking any transmembrane domain (Figure 2A), consistent with its classification as a secreted protein. The protein is notably enriched in serine residues, which comprise approximately 21% of its amino acid composition. In addition, the C-terminal region contains five tandem copies of a conserved 15-amino-acid motif (PVPRAVPVSVPHPVP) (Figure 2A).

Homologous proteins were identified across multiple aphid species (Figure 2B). Sequence comparisons revealed that MsSP1 shares 79.3% amino acid identity with the *Aphis craccivora* homolog (GenBank accession: KAF0769882.1), whereas identity with the *Aphis gossypii* homolog (GenBank accession: CAH1737741.1) was considerably lower at 45.5% (Figure S1). Phylogenetic analysis further demonstrated that MsSP1 clusters most closely with homologs from *Aphis craccivora* and *Rhopalosiphum padi*, forming a distinct evolutionary clade.

### 3.3. MsSP1 Is Delivered into Sorghum Tissues During Aphid Feeding

**Figure 3:**
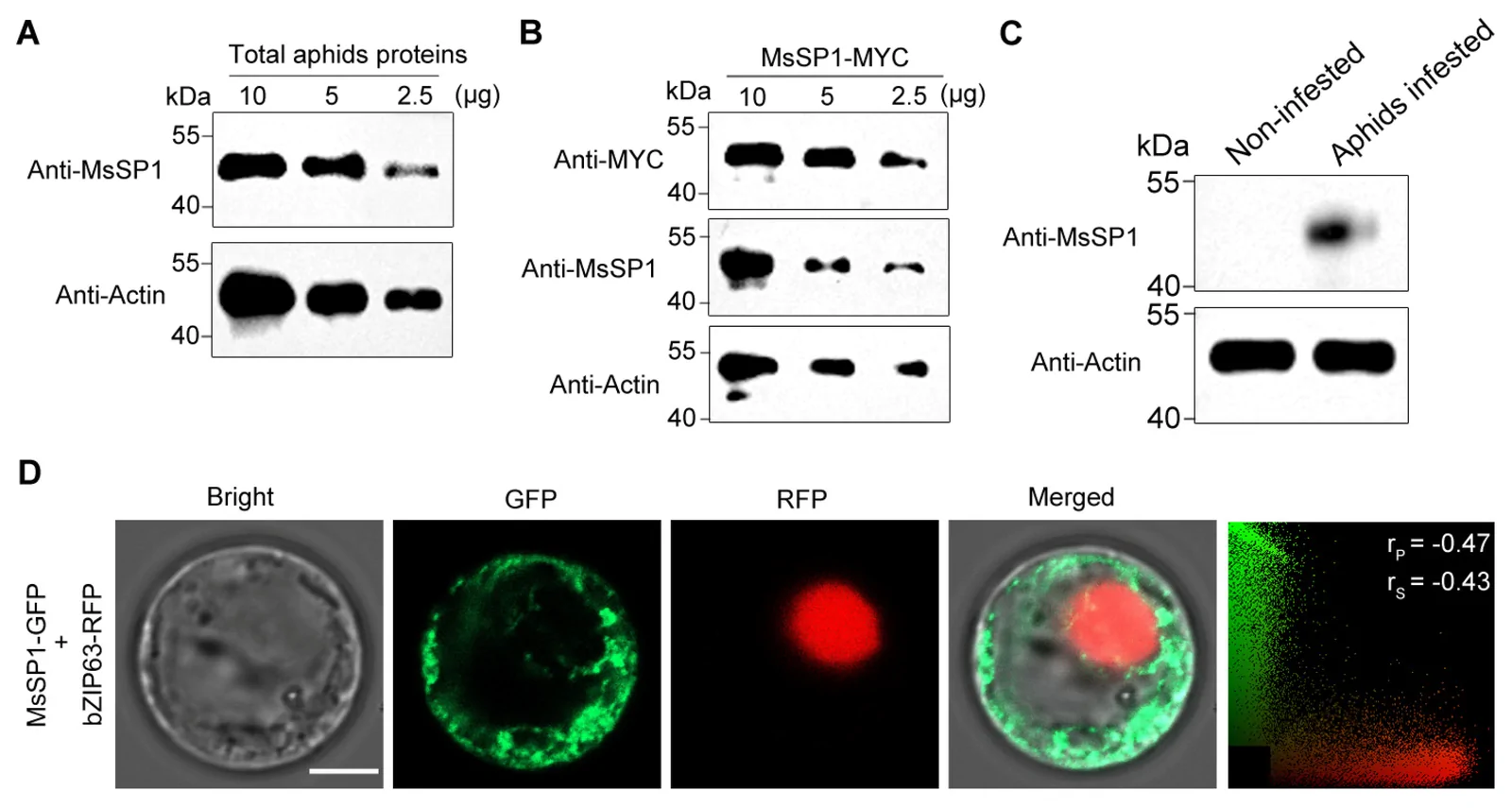
MsSP1 is delivered into sorghum tissues during aphid feeding. (**A**) Detection of endogenous MsSP1 in *M. sorghi* using serial dilutions (10, 5, and 2.5 μg) of total adult aphid proteins. (**B**) The specificity of the anti-MsSP1 antibody was evaluated using transiently expressed MsSP1-MYC protein in *N. benthamiana*. Gradient-diluted total tobacco proteins (10, 5, and 2.5 μg) were detected with anti-MsSP1 or anti-MYC antibodies. (**C**) Detection of MsSP1 in sorghum leaves following aphid infestation. Total proteins extracted from aphid-infested and non-infested sorghum seedlings were analyzed by immunoblotting with the anti-MsSP1 antibody. In (**A**–**C**), anti-Actin immunoblotting served as the loading control. (**D**) Subcellular localization of MsSP1 in sorghum protoplasts. bZIP63-RFP was used as a nuclear marker. The values of the Pearson correlation coefficient (r_P_) and Spearman correlation coefficients (r_S_) range from −1 to +1, representing a strong negative correlation to a strong positive correlation. Scale bar = 5 μm.

To verify whether MsSP1 is secreted into host tissues during aphid feeding, a synthetic peptide (SNAVPVKVSQSDQ) was generated by solid-phase peptide synthesis and used to produce a polyclonal anti-MsSP1 antibody. The specificity of the antibody was first evaluated using total protein extracted from adult sorghum aphids. Immunoblot analysis detected a single, distinct protein band corresponding to MsSP1 (Figure 3A). The sorghum and aphid proteins were also probed with the preimmune serum, and the band of MsSP1 was not observed, confirming the high specificity of the MsSP1 antibody (Figure S2). To further validate antibody recognition, MsSP1-MYC was transiently expressed in *N. benthamiana*, and the fusion protein was successfully detected by the anti-MsSP1 antibody (Figure 3B).

To determine whether MsSP1 is delivered into plant tissues, sorghum aphids were allowed to feed on BTx623 plants for 48 h. Immunoblot analysis detected MsSP1 in protein extracts from aphid-infested leaf sheaths but not in uninfested controls (Figure 3C), demonstrating that the protein is secreted into sorghum tissues during feeding.

The subcellular localization of MsSP1 was subsequently examined in sorghum protoplasts. Co-expression of MsSP1-GFP with the nuclear marker bZIP63-RFP revealed that GFP fluorescence was restricted to the cytoplasm and showed no overlap with the nuclear signal (Figure 3D), indicating that MsSP1 localizes predominantly to the cytoplasmic compartment.

### 3.4. Silencing MsSP1 Reduces Aphid Reproductive Performance

**Figure 4:**
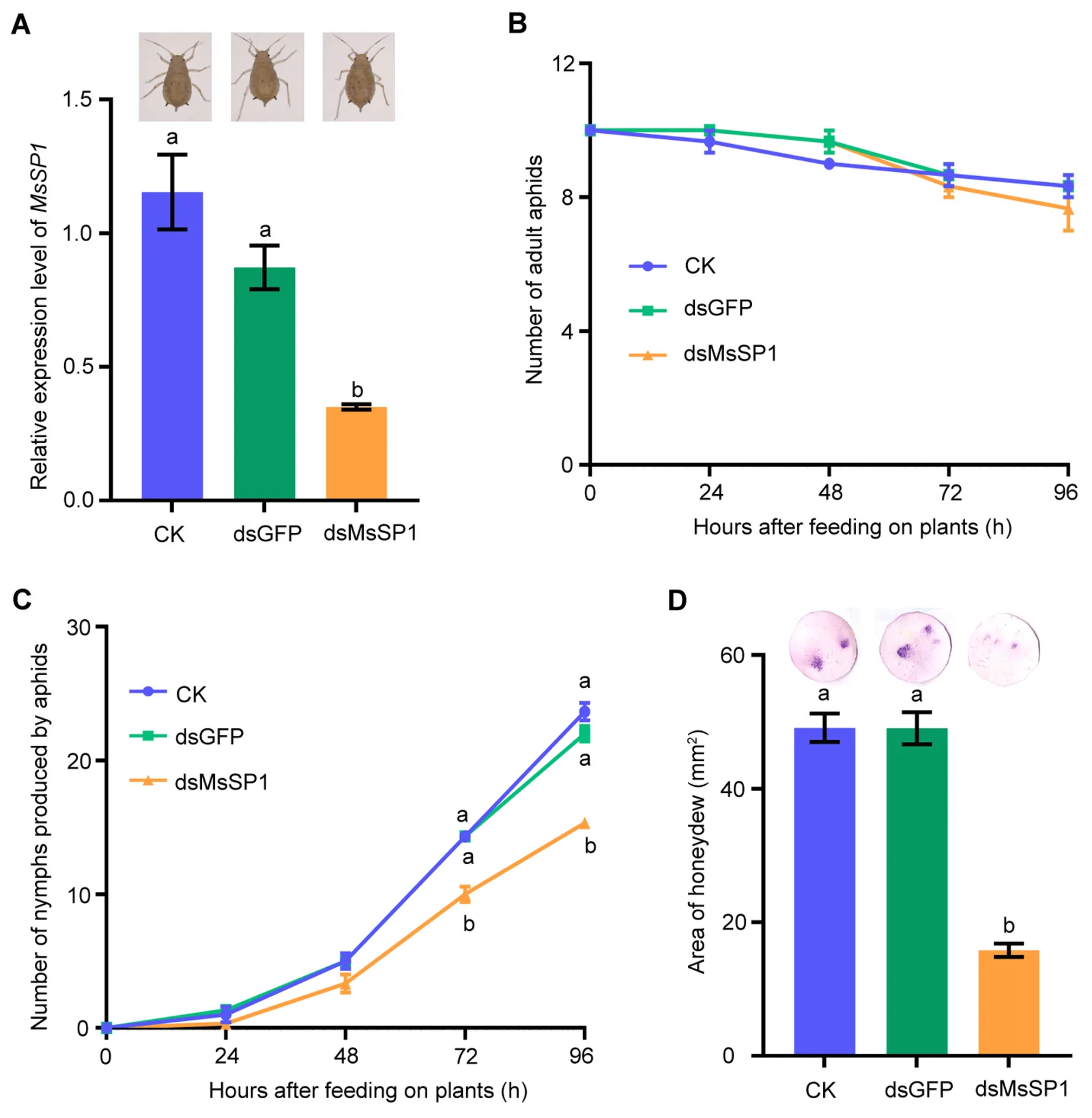
Effects of *MsSP1* silencing on *M. sorghi* feeding and performance. (**A**) RNA interference efficiency was evaluated by measuring *MsSP1* transcript abundance. No visible morphological alterations were observed following gene silencing. Data represent means ± SE of three independent biological replicates, each with three technical replicates. (**B**) Survival of aphids was monitored daily after treatment. (**C**) Total nymph production was recorded over time. Aphids treated with dsMsSP1 produced significantly fewer offspring than those in either control group. (**B**,**C**) Data represent means ± SE of three independent experiments with 10 aphids per replicate. (**D**) Honeydew excretion on filter paper. The intensity and area of staining reflect aphid feeding activity. CK, aphids without dsRNA treatment; dsGFP, aphids fed GFP dsRNA; dsMsSP1, aphids fed *MsSP1* dsRNA. Data represent means ± SE of three biological replicates with 5 filter papers per replicate. Different letters indicate significant differences according to Tukey’s HSD test (*p* < 0.05).

To investigate the biological function of MsSP1 in *M. sorghi*, RNA interference was performed by feeding aphid nymphs double-stranded RNA targeting *MsSP1* (dsMsSP1). Quantitative RT-PCR confirmed effective gene silencing, with *MsSP1* transcript levels significantly reduced at 48 h after dsRNA treatment compared with untreated aphids (CK) and aphids fed dsGFP (Figure 4A). Following transfer to BTx623 plants, *MsSP1*-silenced aphids displayed no obvious morphological abnormalities and showed survival rates comparable to those of both control groups (Figure 4A,B). In contrast, aphid reproductive output was markedly reduced. Aphids treated with dsMsSP1 produced significantly fewer offspring than untreated or dsGFP-fed controls (Figure 4C). Consistent with reduced feeding activity, dsMsSP1-treated aphids also excreted substantially less honeydew than control aphids feeding on the same host plants (Figure 4D). Together, these results indicate that *MsSP1* contributes to aphid feeding efficiency and reproductive fitness on sorghum.

### 3.5. MsSP1 Suppresses Plant Immune Responses

**Figure 5:**
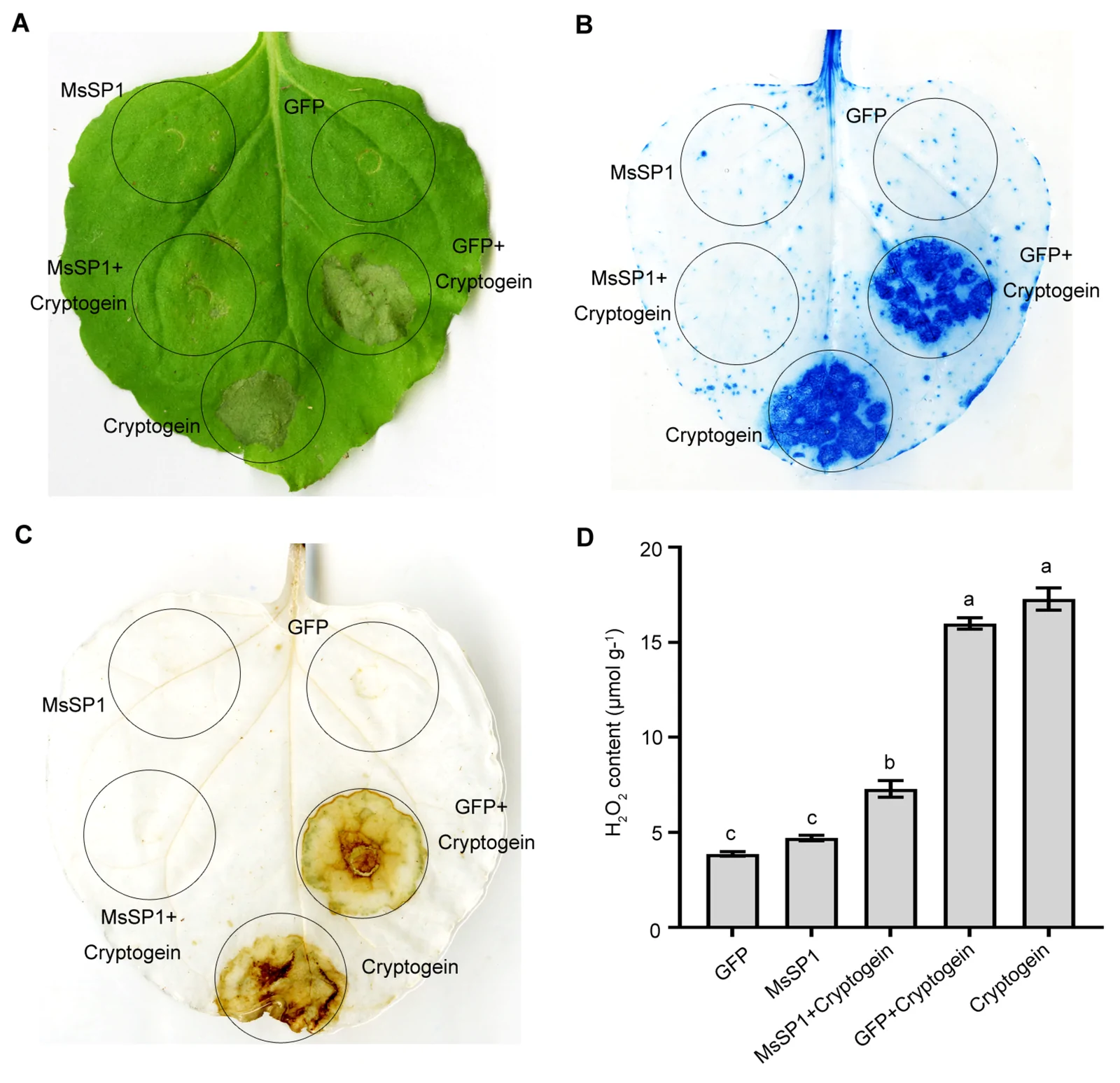
MsSP1 suppresses cryptogein-induced cell death and ROS accumulation in *N. benthamiana*. (**A**,**B**) Cell death was assessed by visual observation (**A**) and trypan blue staining (**B**). Cryptogein, a known inducer of plant cell death [47], was used as a positive control. Co-expression of MsSP1 with cryptogein markedly reduced cell death symptoms. Photographs were taken 5 days after infiltration. (**C**,**D**) DAB staining (**C**) and quantitative measurement of H_2_O_2_ content (**D**) showed that MsSP1 suppresses cryptogein-induced ROS accumulation. GFP served as a negative control. Different letters indicated significant differences according to Tukey’s HSD test (*p* < 0.05). Values represented means ± SE from three biological replicates, each with three technical replicates.

The observation that MsSP1 is delivered into sorghum tissues (Figure 3C) and accumulates in the cytoplasm of plant cells (Figure 3D) prompted us to examine its potential role in plant immunity. To this end, a signal peptide-truncated MsSP1 construct was transiently expressed in *N. benthamiana* via *Agrobacterium*-mediated transformation. Cryptogein, a well-characterized elicitor from *Phytophthora cryptogea* that triggers reactive oxygen species (ROS) production and hypersensitive response (HR)-associated cell death in *N. benthamiana* [46], was used as a positive control, whereas GFP served as a negative control. Recombinant constructs expressing MsSP1-MYC, GFP-MYC, and Cryptogein-FLAG under the control of the CaMV 35S promoter were infiltrated into tobacco leaves. Immunoblot analysis confirmed comparable accumulation of all recombinant proteins 48 h after infiltration (Figure S3). As expected, expression of Cryptogein-FLAG alone induced pronounced chlorosis and necrosis. In contrast, co-expression of MsSP1-MYC markedly attenuated cryptogein-induced symptoms, whereas expression of GFP or MsSP1 alone did not cause visible tissue damage (Figure 5A). Trypan blue staining yielded similar results (Figure 5B), and ion leakage was significantly lower from leaves co-expressing MsSP1 and cryptogein than from the cryptogein-expressing leaves alone, further demonstrating suppression of cryptogein-triggered cell death by MsSP1 (Figure S4).

To determine whether MsSP1 also affects ROS production, DAB staining was performed to visualize hydrogen peroxide accumulation. Strong brown staining was observed in tissues expressing cryptogein alone, whereas staining was greatly reduced when cryptogein was co-expressed with MsSP1 (Figure 5C). Consistent with these observations, quantitative measurements showed significantly lower H_2_O_2_ levels in leaves co-expressing MsSP1 and cryptogein than in leaves expressing cryptogein alone (Figure 5D). The reduction in H_2_O_2_ accumulation was also detected in sorghum protoplasts co-expressing cryptogein and MsSP1 (Figure S5). Notably, expression of MsSP1 by itself neither induced H_2_O_2_ accumulation nor triggered cell death, indicating that its primary function is to suppress plant immune responses rather than activate them.

## 4. Discussion

Although substantial progress has been made in characterizing salivary and secreted proteins from piercing-sucking insects, including the brown planthopper (*Nilaparvata lugens*) [9], green peach aphid (*Myzus persicae*) [48], potato aphid (*Macrosiphum euphorbiae*) [49], and whitefly (*Bemisia tabaci*) [50], knowledge of the secreted proteins of the sorghum aphid remains limited, particularly regarding proteins involved in host immune manipulation. In the present study, we combined proteomic and functional analysis to characterize secreted proteins from *M. sorghi* and explore their potential roles in aphid–sorghum interactions. Our findings suggest that MsSP1 proteins may function as candidate effectors that facilitate aphid adaptation to sorghum while simultaneously manipulating plant defense, thereby contributing to the ongoing coevolution between sorghum and its aphid pests.

The composition of the *M. sorghi*-derived proteins detected in aphid-infested sorghum tissues showed considerable overlap with secretory proteins reported in other herbivorous arthropods. Multiple protein classes identified in this study, including metabolic enzymes such as malate dehydrogenase, UDP-glucose pyrophosphorylase, and glutamine synthetase, as well as regulatory proteins, RNA-binding proteins, molecular chaperones, and ribosomal proteins, have also been detected in the secretomes of *Nilaparvata lugens*, *Acyrthosiphon pisum*, and *Tetranychus urticae* [9,51,52]. This conservation across species suggests that many secreted proteins perform fundamental functions that have been maintained during arthropod evolution.

Secreted proteins delivered into plant tissues are widely recognized as important mediators of herbivore–host interactions [11,13,17,18]. By integrating proteomic data with signal peptide prediction, we identified five candidate secreted proteins containing N-terminal secretion signals among the 69 *M. sorghi*-derived proteins. Subsequent immunoblot analysis confirmed that one of these proteins, MsSP1, is delivered into sorghum tissues during aphid feeding, although further evidence, such as immunolocalization around feeding sites, will be developed in the future. Notably, MsSP1 contains abundant serine residues and multiple tandem-repeat motifs. Similar Ser/Thr-rich domains are common in fungal secreted proteins and often serve as sites of extensive O-glycosylation associated with extracellular stability, virulence, and host interactions [53]. The abundance of serine residues in the *Nilaparvata lugens* salivary protein NlMLP is reported to provide multiple carbohydrate determinants that lead to the formation of extracellular salivary sheaths facilitating insect feeding [39]. So, it appears that these serine residues in the MsSP1 protein may play similar roles. Functional analysis further demonstrated that silencing *MsSP1* significantly reduced aphid feeding activity and fecundity, indicating an important role in aphid fitness and host colonization. However, *MsSP1*-deficient aphids fed on sorghum plants displayed normal survival; we speculate that silencing *MsSP1* in sorghum aphids has no effect on fat storage or energy mobilization within a short time, which are critical parameters for insect survival [54,55].

Plants rapidly perceive insect feeding through both mechanical damage and salivary components, triggering defense responses that limit herbivore performance. In turn, herbivorous insects deploy effectors that suppress these defenses and promote successful infestation [18,56,57,58,59]. For example, Mp1, Me10, Me23, and MpC002 enhance the longevity and reproduction of aphids, and MpMIF, Mp 55, and Mp10 suppress callose and ROS accumulation in plants [11,17,18,19,29]. Consistent with these known effectors, transient expression of MsSP1 in sorghum protoplasts and *Nicotiana benthamiana* markedly suppressed cryptogein-induced ROS accumulation and cell death. These findings support the hypothesis that MsSP1 functions as a virulence effector that attenuates plant immune responses and thereby enhances aphid performance.

Potential functions of insect secreted proteins are often inferred from homologous proteins previously associated with parasitism or host manipulation in other piercing-sucking insects [56,60,61]. In addition to MsSP1, four other signal peptide-containing proteins were identified in the proteomic analysis: a maltase A1-like protein (XP_025200270.1), a lysine-rich arabinogalactan-like protein (XP_025203063.1), and two other unannotated proteins (XP_025201474.1 and XP_025204390.1). Members of the maltase family contribute to carbohydrate digestion, survival, and host infestation in insects [62,63,64]. Arabinogalactan proteins have been implicated in diverse biological processes, including cell proliferation, programmed cell death, and hormone-mediated signaling [65,66]. In addition, unannotated proteins are frequently important effector candidates. The uncharacteristic protein c002 from *Acyrthosiphon pisum* was found to be an important effector that is crucial in aphid feeding and interaction with host plants [17]. Therefore, further work is needed to validate whether other signal-peptide-containing proteins may play roles in activating or inhibiting plant defense.

Importantly, the absence of a signal peptide does not necessarily preclude secretion. Increasing evidence indicates that many proteins are exported through non-classical secretory pathways and can influence herbivore performance or host immunity despite lacking canonical secretion signals [67,68]. Several such proteins were detected in the *M. sorghi*-derived proteins in the proteomic analysis by SecretomeP prediction (Table S1). In addition, glutathione S-transferase-like protein (XP_025201982.1), glutamine synthetase 2 (XP_025202478.1), and myrosinase 1-like protein (XP_025206927.1) may also participate in counteracting the plant defense system. Glutathione S-transferases and glutamine synthetases are key components of detoxification, stress tolerance, immune regulation, and reproductive processes in insects [69,70,71]. In particular, glutathione S-transferases facilitate insect adaptation to host-derived secondary metabolites through antioxidant activity, detoxification, and ligand-binding functions that can mitigate plant defense responses [71]. Myrosinase represents another intriguing candidate. In plants, myrosinases are typically localized in vacuoles and hydrolyze glucosinolates into toxic defense compounds following herbivore attack [72]. Conversely, aphids can exploit glucosinolates together with endogenous myrosinase activity to evade host defenses and enhance their own protection [73]. Further investigation of these proteins may provide important insights into the molecular strategies used by sorghum aphids to circumvent plant immunity.

Recognition of herbivorous insects by plants is frequently accompanied by ROS production and regulated cell death, both of which contribute to defense activation [74]. Numerous studies have demonstrated that ROS accumulation occurs in vascular tissues during biotic and abiotic stress and is involved in cell wall remodeling and defense signaling processes, thus negatively affecting phloem feeding by sap-sucking insects [75,76,77]. In the present study, MsSP1 strongly suppressed cryptogein-induced ROS accumulation and cell death, and affected aphid feeding performance in host plants. It appears that sorghum aphids have the ability to inhibit ROS accumulation in plants’ vascular tissues by continuously secreting MsSP1 proteins, thereby dampening host defense signaling and enhancing their feeding in the phloem. Identifying the direct molecular targets of MsSP1 and verifying the protein–protein interaction in sorghum cells will be an important objective for future research and may reveal how this effector interferes with downstream immune pathways.

Interestingly, the resistant sorghum genotype HN16 employs the resistance genes *RMES1A* and *RMES1B* to activate robust defense responses, including ROS bursts and callose deposition, which restrict aphid feeding, development, and reproduction while maintaining normal plant growth [78]. These findings raise the possibility that resistant sorghum varieties have evolved mechanisms capable of counteracting effector activity such as those of MsSP1. Understanding how host resistance overcomes aphid effectors may provide valuable opportunities for the development of durable aphid-resistant sorghum cultivars.

## 5. Conclusions

Using a proteomics-based approach, we identified 69 *M. sorghi*-derived proteins detected in aphid-infested sorghum tissues. Bioinformatic analyses predicted signal peptides in five of these proteins, highlighting them as potential secreted candidates. Among them, MsSP1 was experimentally confirmed to be delivered into sorghum tissues during aphid feeding. Functional characterization demonstrated that MsSP1 contributes to aphid feeding activity and fecundity while suppressing cryptogein-induced immune responses in plants. Collectively, these findings provide a promising candidate effector, MsSP1, for RNAi-based pest control strategies. Further investigation to identify the direct targets of MsSP1 in sorghum will be conducive to clarifying the molecular mechanism of aphid–sorghum interaction.

## Data Availability

Please contact the corresponding author upon reasonable request.

## Supplementary Materials

The following supporting information can be downloaded at https://www.mdpi.com/article/10.3390/insects17080779/s1: Figure S1: Multiple sequence alignment of MsSP1 and its orthologs from six types of aphids; Figure S2. The preimmune serum does not react with in sorghum and sorghum aphids; Figure S3: Immunoblot analysis of MsSP1-MYC, GFP-MYC, and Cryptogein-FLAG accumulation in *N. benthamiana* leaves 48 h after agroinfiltration; Figure S4. The measurement of electrolyte leakage in *N. benthamiana*; Figure S5. MsSP1 suppresses cryptogein-triggered H_2_O_2_ burst in sorghum protoplast; Table S1: Detailed information for all 69 *M. sorghi*-derived proteins; Table S2: Gene Ontology biological process of *M. sorghi*-derived proteins; Table S3: KEGG pathway analysis of *M. sorghi*-derived proteins; Table S4: The list of primers used in this study.
