# Supplementary material for: Identification and Functional Analysis of Candidate Effectors in the Sorghum Aphid, Melanaphis sorghi
Source: Insects. 2026 Jul 28;17(8):779. doi: 10.3390/insects17080779 (PMC13513276; doi:10.3390/insects17080779)
Supplement: Supplementary file 1 [file insects-17-00779-s001.zip › R1-Insects-Supplementary Figures.pdf]

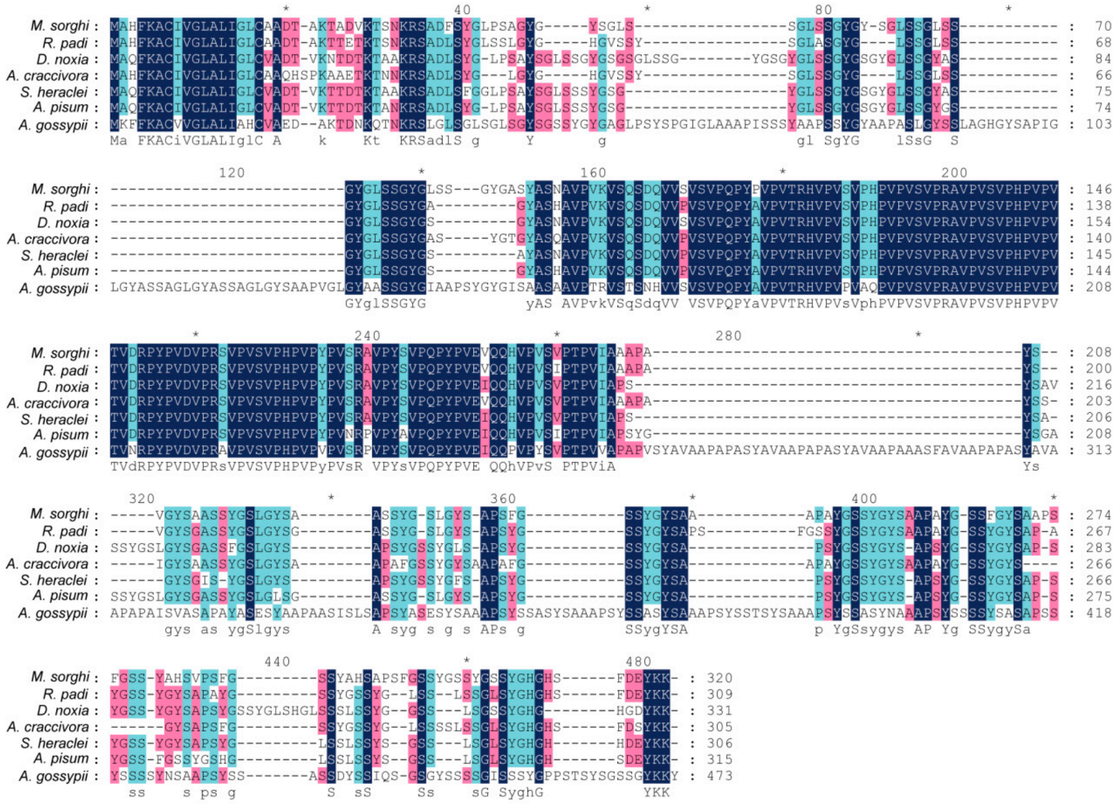

Figure S1. Multiple sequence alignment of MsSP1 and its orthologs from six types of aphids. *R. padi* (XP_060844876.1), *D. noxia* (XP_015374195.1), *A. craccivora* (KAF0769882.1), *S. heraclei* (KAL5237269.1), *A. pisum* (NP_001313557.1), and *A. gossypii* (CAH1737741.1). Black, red, and blue shading indicate 100%, >75%, and 50% amino acid identity, respectively.

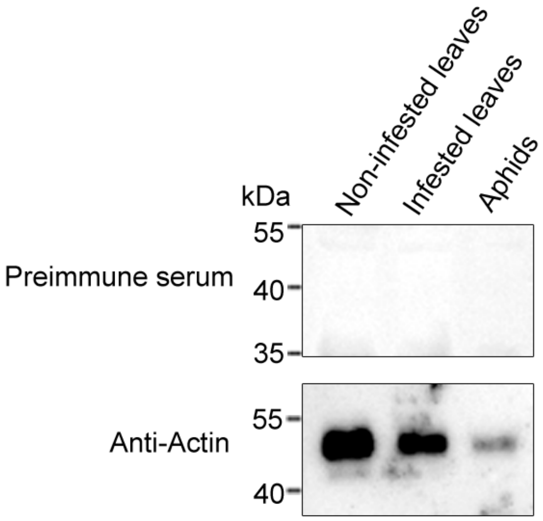

Figure S2. The preimmune serum does not react with in sorghum and sorghum aphids. The total proteins were extracted from infested or non-infested sorghum leaves and aphids and analyzed by immunoblotting with the preimmune serum. anti-Actin immunoblotting served as the loading control.

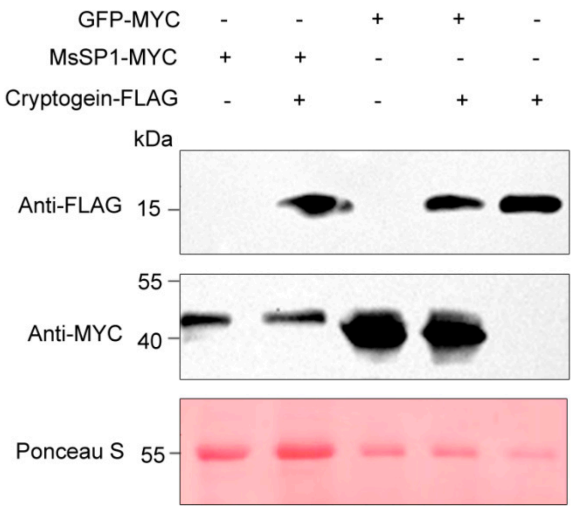

Figure S3. Immunoblot analysis of MsSP1-MYC, GFP-MYC, and Cryptogein-FLAG accumulation in *N. benthamiana* leaves 48 hours after agroinfiltration. The five treatments were MsSP1-MYC alone, MsSP1-MYC + Cryptogein-FLAG, GFP-MYC alone, GFP-MYC + Cryptogein-FLAG, and Cryptogein-FLAG alone.

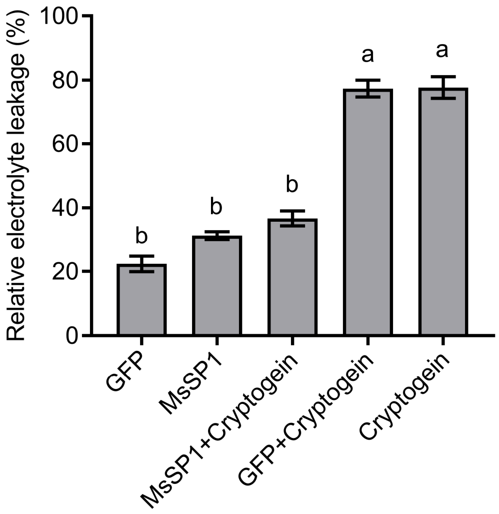

Figure S4. The measurement of electrolyte leakage in *N. benthamiana*. Relative electrolyte leakage was calculated after leaf discs were infiltrated by *Agrobacterium* for 36 hours. Data represent means ± SE of three repeats. Different letters indicated significant differences according to Tukey's HSD test ($P < 0.05$).

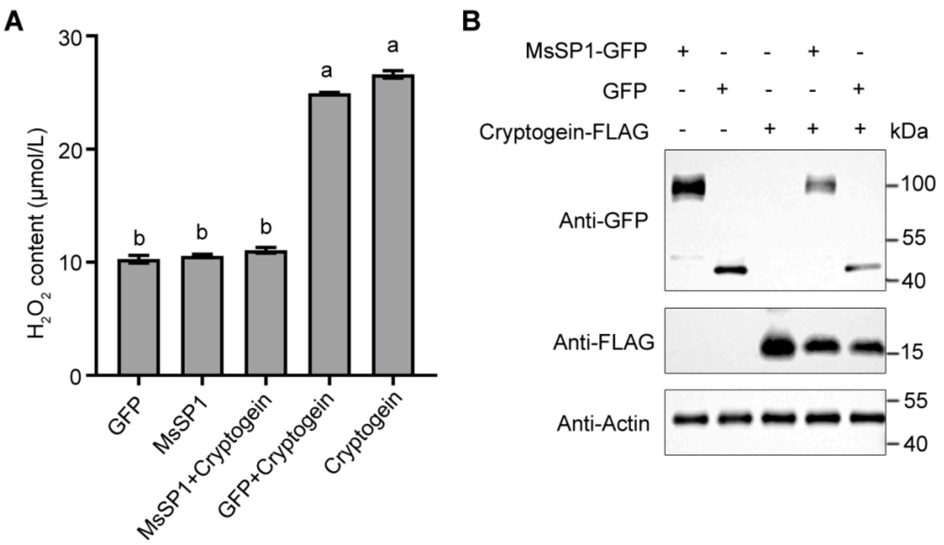

Figure S5. MsSP1 suppresses cryptogein-triggered H_2O_2 burst in sorghum protoplast. (A) Quantitative comparison of H_2O_2 contents in sorghum protoplast co-expressing different pairs of constructs. GFP and Cryptogein served as negative and positive control, respectively. The H_2O_2 content was measured at 24 h after protoplast transfection. Different letters indicated significant differences according to Tukey's HSD test ($P < 0.05$). Values represented means ± SE from three biological replicates, each with three technical replicates. (B) Immunoblotting detection the expression of MsSP1-GFP, Cryptogein-FLAG, and GFP in the transfected sorghum protoplast.
